# Whole genome sequences of three Clade 3 *Clostridium difficile* strains carrying binary toxin genes in China

**DOI:** 10.1038/srep43555

**Published:** 2017-03-06

**Authors:** Rong Chen, Yu Feng, Xiaohui Wang, Jingyu Yang, Xiaoxia Zhang, Xiaoju Lü, Zhiyong Zong

**Affiliations:** 1 Center of Infectious Diseases, West China Hospital, Sichuan University, Chengdu, China; 2 Division of Infectious Diseases, State Key Laboratory of Biotherapy, Chengdu, China; 3 Department of Infection Control, West China Hospital, Sichuan University, Chengdu, China

## Abstract

*Clostridium difficile* consists of six clades but studies on Clade 3 are limited. Here, we report genome sequences of three Clade 3 *C. difficile* strains carrying genes encoding toxin A and B and the binary toxin. Isolates 103 and 133 (both of ST5) and isolate 106 (ST285) were recovered from three ICU patients. Whole genome sequencing using HiSeq 2500 revealed 4.1-Mb genomes with 28–29% GC content. There were ≥1,104 SNP between the isolates, suggesting they were not of a single clone. The toxin A and B gene-carrying pathogenicity locus (PaLoc) of the three isolates were identical and had the insertion of the transposon Tn*6218*. The genetic components of PaLoc among Clade 3 strains were the same with only a few nucleotide mutations and deletions/insertions, suggesting that the Tn*6218* insertion might have occurred before the divergence within Clade 3. The binary toxin-genes carrying CDT locus (CdtLoc) of the three isolates were identical and were highly similar to those of other Clade 3 strains, but were more divergent from those of other clades. In conclusion, Clade 3 has an unusual clade-specific PaLoc characteristic of a Tn*6218* insertion which appears to be the main feature to distinguish Clade 3 from other *C. difficile*.

*Clostridium difficile*, a Gram-positive spore-forming anaerobic bacterium, is the leading cause of antibiotic-associated diarrhea^1^ and also causes colitis which is not necessarily associated with antibiotic use^2^,^3^. Clinical manifestations of *C. difficile* infection (CDI) range between asymptomatic and mild or severe diarrhea which can even lead to death^2^,^3^. The main virulent factors of *C. difficile* are toxin A (enterotoxin, 308 kD) and toxin B (cytotoxin; 270 kD) encoded by genes *tcdA* and *tcdB*, respectively. These two genes together with three regulator genes *tcdC* (a negative regulator), *tcdE* (encoding a bacteriophage-like putative holin to facilitate the release of toxin A and B) and *tcdR* (encoding an alternative σ factor) are located on a pathogenicity locus called PaLoc (Fig. 1)^4^. PaLoc is typically 19.6 kb in length and is almost always integrated at the same location between genes *cdu1* (encoding a transcriptional terminator) and *cdd1* (encoding a putative ABC transporter) in the *C. difficile* genome. By contrast, in non-toxingenic strains such as CD37, the PaLoc is absent and there is a 115-bp non-coding sequence in its place (Fig. 1). At present, *C. difficile* consists of six distinct phylogenetic clades designated Clade 1, 2, 3, 4, 5 and C-I^5^,^6^ and the genetic composition of PaLoc appears to be clade-specific^5^. Among the six clades, Clade 3 has received relatively little interest. Currently, isolates of Clade 3 have been found to belong to ST5 (PCR ribotype 023 or 069), ST22 (PCR ribotype 023), ST25 (PCR ribotype 023), ST96 (PCR ribotype 058), ST162, ST201 and ST221^5^,^7^. It has been suggested that PCR ribotype 023 is able to cause CDI with the same severity of illness as PCR ribotype 027^8^, which is a well-characterized hypervirulent lineage causing outbreaks and severe illness such as toxic megacolon^9^. Nonetheless, although the infection caused by strains of PCR ribotype 023 is associated with high levels of biomarkers such as white blood cell count and C-reactive protein, the 14-day mortality (7%) in CDI cases caused by PCR ribotype 023 was lower than those (12–25%) caused by PCR ribotypes belonging to clade 1, 2, 4 and 5^10^. PCR ribotype 023 is one of the most common ribotypes causing CDI in the world^11^. However, it is not one of the main ribotypes in mainland China^12^ as it only accounts for 0.86% (1/116)^13^ or 2.7% (2/74)^14^ of toxigeneic *C. difficile*.

The PaLoc of Clade 3 is unusual as a transposon. Tn*6218* is inserted between genes *tcdE* and *tcdA*, which results in a larger PaLoc^5^ (28.6 kb; Fig. 1). Tn*6218* is related to the well-studied conjugative transposon Tn*916* but is non-conjugative^5^. Tn*6218* typically contains four common genes, i.e. *int* (encoding a tyrosine recombinase for catalyzing integration and excision), *xis* (encoding an excisionase), *rep* (encoding a putative topoisomerase as the replication initiation factor) and *xre* (a putative transcription regulatory gene involved in the xenobiotic response), and a variety of accessory genes including antimicrobial resistance determinants^5^.

Binary toxin, the third toxin that has been found to enhance the virulence^15^, is produced by 20% to 32% of *C. difficile* strains according to previous reports^16^,^17^. Genes encoding the binary toxin, *cdtA* and *cdtB*, together with the regulation gene *cdtR* are located on the CDT locus (CdtLoc, Figure S1 in the Supplementary Data)^18^. CdtLoc was distributed among *C. difficile* strains of Clades 2, 3 and 5. Although binary toxin gene-carrying *C. difficile* has been reported worldwide, such isolates remain uncommon in China. Previous studies demonstrated that binary toxin gene-carrying isolates accounted for no more than 7% of all toxigenic *C. difficile*^14^,^19^,^20^,^21^,^22^,^23^,^24^.

During a surveillance project on toxigenic *C. difficile* in our hospital, three Clade 3 isolates were recovered. All of them had genes encoding toxins A and B and the binary toxin. They were then subjected to whole genome sequencing. Here, we report their genome sequence and antimicrobial susceptibilities below.

## Materials and Methods

### Isolates

Three *C. difficile* isolates, 103, 133 and 106, were recovered from three different patients at West China Hospital, Sichuan University, Chengdu, southwest China during a preliminary active screening study of colonization by toxigenic *C. difficile* among ICU patients in 2014. Isolates 103 and 133 were recovered from the rectal swabs of two different patients in the General ICU ward in March and June, respectively, while isolate 106 was recovered from the stool sample of a patient in the Respiratory ICU ward in June. This study was conducted in accordance with the amended Declaration of Helsinki and was approved, under a waiver of consent, by the Ethics Committee of West China Hospital. After an initial alcohol shock treatment to kill vegetative cells, stool specimens were grown on cycloserine-cefoxifin-fructose agar (CCFA; Oxoid, Basingstoke, UK) with 10% sheep blood and incubated 48 h at 37 °C under anaerobic conditions. A single colony of each isolate was further cultured in Brain Heart Infusion (BHI; Oxoid) broth for 24 h. The three isolates were found to produce toxin A and B as determined using the C. DIFF QUIK CHEK COMPLETE enzyme immunoassay kit (Techlab, Blacksburg, VA, USA).

Genomic DNA was prepared from the three isolates using QIAamp DNA Mini Kit (Qiagen, Hilden, Germany). The three isolates were positive to *tcdA, tcdB* and binary toxin genes *cdtA* and *cdtB* by PCR as described previously^25^. The isolates were assigned to sequence types (ST) using the multilocus sequence typing (MLST) scheme targeting seven housekeeping genes, *adk, atpA, dxr, glyA, recA, sodA* and *tpi* (http://pubmlst.org/cdifficile/)^26^ by PCR and Sanger sequencing, which was also confirmed using their genome sequences to query the MLST database (see below).

### Whole genome sequence and phylogenetic analysis

All three isolates were subjected to the 150 bp paired-end whole genome sequencing with a ca. 200× coverage using the HiSeq 2500 Sequencer (Illumina, San Diego, CA, USA) following the manufacturer’s protocol. Reads were filtered using the Sickle quality trimming tool (https://github.com/najoshi/sickle/) with 20 as the cutoff value of both quality and length. Filtered reads were then assembled into contigs using the SPAdes program^27^ with auto-cutoff and careful functions being turned on. Annotation of the genomic sequence was carried out using the Prokka program^28^ against its incorporated bacterial gene database. The coding sequence (CDS) of interest (e.g. unique genes) was then manually checked and modified using Protein BLAST (https://blast.ncbi.nlm.nih.gov/) against non-redundant protein sequences in GenBank. Settings of programs mentioned above, unless specifically noted, all remained as default.

Antimicrobial resistance genes were predicted using the ResFinder web-based tool from the Center for Genomic Epidemiology (http://genomicepidemiology.org/). Prophages were predicted using the PHASTER web server (http://phaster.ca/)^29^. The predicted intact and incomplete phage sequences were searched for similar regions, which were defined as ≥80% coverage and ≥90% highest nucleotide identity, on other *C. difficile* genomes using BLAST (http://blast.ncbi.nlm.nih.gov/).

To identify plasmids, reads filtered by Sickle were assembled into contigs using the PlasmidSPAdes^30^. Contigs assembled by SPAdes and PlasmidSPAdes were screened for plasmids using Microbial Genome BLAST (http://blast.ncbi.nlm.nih.gov/Blast.cgi?PAGE_TYPE=BlastSearch&BLAST_SPEC=MicrobialGenomes) against the complete plasmid database. In addition, sequences of all *C. difficile* plasmids (n = 6; Table S1 in the Supplementary Data) that have been deposited in GenBank were retrieved. Contigs assembled by SPAdes and PlasmidSPAdes of the three isolates in the present study were aligned with the *C. difficile* plasmid sequences using BLAST to further examine the presence of plasmids.

There were four Clade 3 strains with whole genome sequences available in GenBank. These were ZJCDC-S82 (ST5; clinical isolate, recovered in 2013, China, GenBank accession no. JYNK01000000), CD69 (ST221; clinical isolate, recovered in 2010, USA, GenBank accession no. AVHE00000000), VL-0391 (ST201; clinical isolate, recovered date not available, Canada, GenBank accession no. FALK01000001) and VL-0104 (ST201; clinical isolate, recovered date not available, Canada, GenBank accession no. FAAJ0100000). In addition, six strains of Clade 3 from the UK (Oxford) and Australia have been reported^5^ with short reads of genome sequences available in the Sequence Read Achieve (SRA) database. The short reads of these strains were retrieved and were assembled into contigs using the SPAdes program^27^, which were then used for comparison. All of the Clade 3 strains listed above carry genes encoding toxins A and B and the binary toxin.

Strains with whole genome sequence available in GenBank (http://www.ncbi.nlm.nih.gov/genome/genomes/535?, accessed by July 31, 2016) that belong to a Clade other than Clade 3 could be assigned to 44 ST. Whole genome sequences of all Clade 3 strains (n = 13; 10 from GenBank and SRA databases and 3 in the present study) and a representative strain of each of the 44 ST of other clades (Table 1) were included into the phylogenetic analysis. The representative genomes selected, were based on those that have been included in analyses to infer the phylogeny of *C. difficile* in two previous studies^31^,^32^. The genomes of 57 strains were aligned using the Harvest suite^33^ with the incorporated Phi test, which identifies recombination sites, being turned on and other settings remaining as default. Approximately 2 Mb sequences representing the core genome of *C. difficile* were identified. After removing single nucleotide polymorphisms (SNP) either on a recombination site or not tagged with ‘PASS’ using Harvest^33^, a total of 180,004 SNP sites in the core genome were kept for further analyses. The phylogenetic tree was inferred based on all filtered SNP by MEGA 7.0^34^ using the neighbour-joining method with Jukes-Cantor and 1,000 bootstrap replicates as the nucleotide substitution model and method to test phylogeny, respectively. A circular plot was constructed using the Circleator tool^35^ to demonstrate the locations of SNPs among the three isolates in the present study. Pair-wise average nucleotide identity (ANI) based on BLAST was calculated for the genome sequence of the three isolates and all Clade 3 strains and the 44 representative strains of other clades (Table 1) using the JSpecies web program (http://imedea.uib-csic.es/jspecies/) with default settings^36^.

The pan-genomes of 13 strains of Clade 3 were constructed based on the amino acids of previously predicted genes (5,656 genes in total) and were then aligned against 44 annotated representative genomes of other clades (Table 1) by Roary^37^ with default settings. Genes present in all of 13 strains of Clade 3 but absent (arbitrarily defined as <80% nucleotide identity and <70% coverage) from strains of other clades were further examined whether they were unique to Clade 3 among *C. difficile* using Microbial Genome BLAST against all available *C. difficile* genomes. Genes present in only one of the three isolates were considered unique. This was also confirmed using Microbial Genome BLAST against all *C. difficile* genomes. The function of genes that was unique to one of the three isolates or Clade 3 was further predicted using Protein BLAST. The unique regions were searched for insertion sequences using ISFinder (https://www-is.biotoul.fr/) and integrative and conjugative elements (ICE) using ICEberg (http://db-mml.sjtu.edu.cn/ICEberg/).

The sequence of PaLoc and CdtLoc of the three isolates were aligned to genomes of all Clade 3 strains and representative strains of other clades. A phylogenetic tree of CdtLoc was constructed for all Clade 3 strains and several representative strains of Clade 2 or Clade 5 using the Harvest tool^33^.

### Antimicrobial susceptibility testing

Minimum inhibitor concentrations (MICs) of clindamycin, fidaxomicin, metronidazole, moxifloxacin, rifampicin, tetracycline and vancomycin were determined using the agar dilution method following the recommendations of the Clinical and Laboratory Standard Institute (CLSI) guidelines^38^. Breakpoints defined by CLSI (clindamycin, fidaxomicin, metronidazole, moxifloxacin, rifampicin and tetracycline) or by the European Committee on Antimicrobial Susceptibility Testing (EUCAST; vancomycin) were applied.

## Results

### *C. difficile* isolates, MLST and general genome features

Isolates 103 and 133 belonged to ST5 (*adk1, atpA6, dxr4, glyA7, recA2, sodA8*, and *tpi7*), while the remaining isolate, 106, belonged to ST285 (*adk1, atpA6, dxr4, glyA7, recA25, sodA8*, and *tpi7*). ST5 and ST285 differ in a single allele (*recA*) with only one SNP. A total of 5,703,662 to 5,812,358 reads were generated by whole genome sequencing of the three isolates. These were assembled into 243, 303 and 276 contigs (61, 69 and 75 contigs ≥1,000 bp in length; N50, 131,308 to 137,573 bp) with a 28.38 to 28.74% GC content, respectively (Table 2). There were 3,764 to 4,000 CDS that were identified from the genome of the three isolates. Of note, isolate 133 had 227 or 236 more CDS than the other two isolates, which is due to a 150 kb phage present on 133 but absent from the other two (see below).

### SNP and phylogenetic analysis

There were 1,267 SNPs between isolate 103 and 133 (both of ST5), while isolate 106 (ST285) had 1,364 and 1,104 SNPs compared with isolate 133 and 103, respectively (Figure S2 in the Supplementary Data). The phylogenetic tree constructed correlates well to the distribution of clades (Fig. 2, the number of SNPs is listed in Table S3 in the Supplementary Data). All Clade 3 strains were clustered together and were closer to Clade 4, 5 and C-I rather than Clade 1 and 2 (Fig. 2). In Clade 3, isolates 106 and 133 were clustered with strain CD69, while isolate 103 appeared to be more distinct from isolates 103 and 106 (Fig. 3).

When compared with other Clade 3 strains and 44 representative strains of each clade, isolate 103 had the highest ANI value with isolates 106 (99.52%) and 133 (99.51%; Table 1). The ANI values between any two strains of Clade 3 were all above 99%, while those between a Clade 3 strain and strains of Clade 1, 2, 4 and 5 were in the range of 96.3% to 98.1%. The ANI value between Clade 3 and Clade C-I was only 90% to 91.4%.

### Antimicrobial susceptibility and antimicrobial resistance genes

All three isolates were susceptible to clindamycin (MIC, 0.125 to 2 μg/mL), fidaxomicin (0.015 to 0.06 μg/mL), metronidazole (<0.03 μg/mL), moxifloxacin (<0.03 to 2 μg/mL), rifampicin (<0.03 μg/mL), tetracycline (<0.03 to 0.06 μg/mL) and vancomycin (<0.03 to 0.25 μg/mL). Correspondingly, no known antimicrobial resistance genes were identified from their genome sequences.

### Sequence analysis of PaLoc

**Figure 1:**
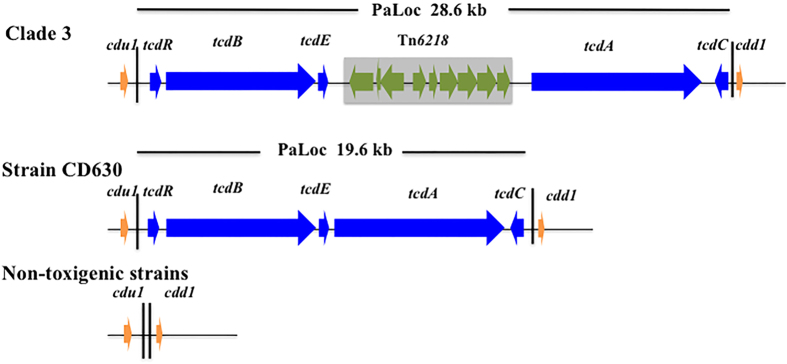
Schematic representations of PaLoc and flanking genes. The 5′ flanking gene *cdu1*, the 3′ flanking gene *cdd1*, positive regulator gene *tcdR*, negative regulator gene *tcdC*, and toxin genes *tcdA* and *tcdB* are shown. For Tn*6218*, the genetic components from left to right are *int, xis, rep, xre, merR*, oxidoreductase-encoding gene, flavodoxin-encoding gene, orf and σ70-encoding gene.

**Figure 2:**
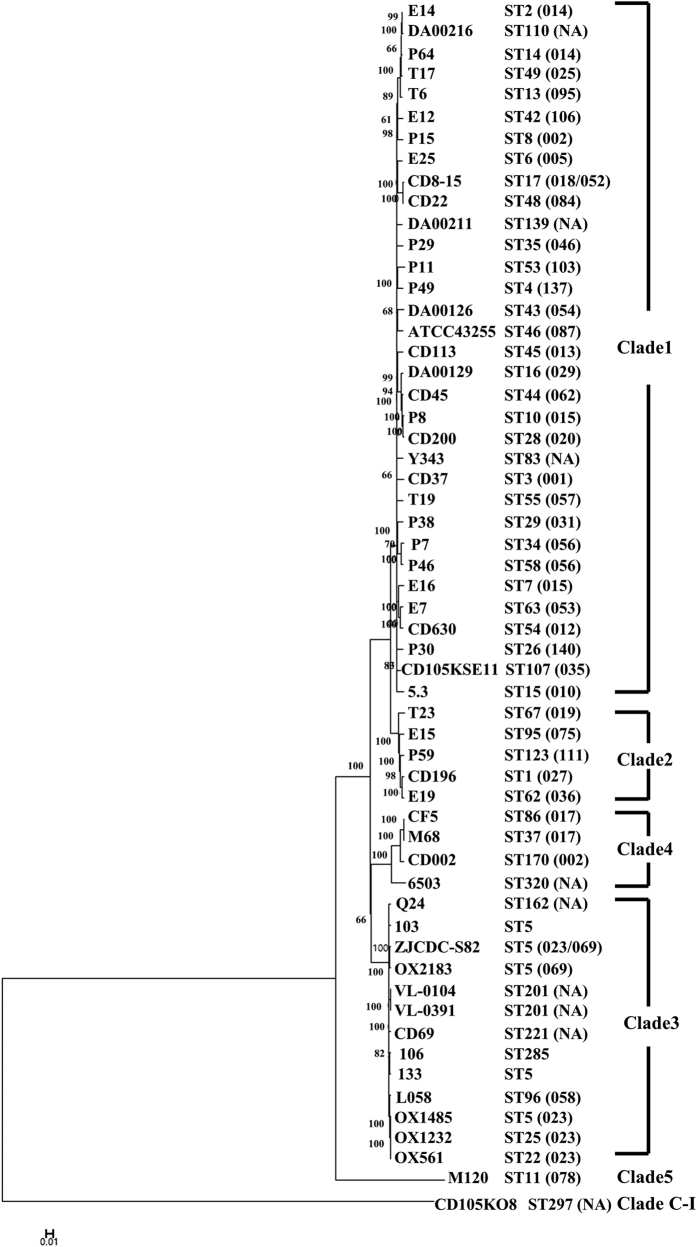
Phylogenetic tree based on SNPs of genome sequences of the three isolates, other 10 strains of Clade 3 and 44 representative strains of other clades. ST is shown after the strain names and PCR ribotype is indicated in parentheses with NA representing not available. The three isolates are highlighted with boxes. Bar, 0.02 changes per nucleotide position.

**Figure 3:**
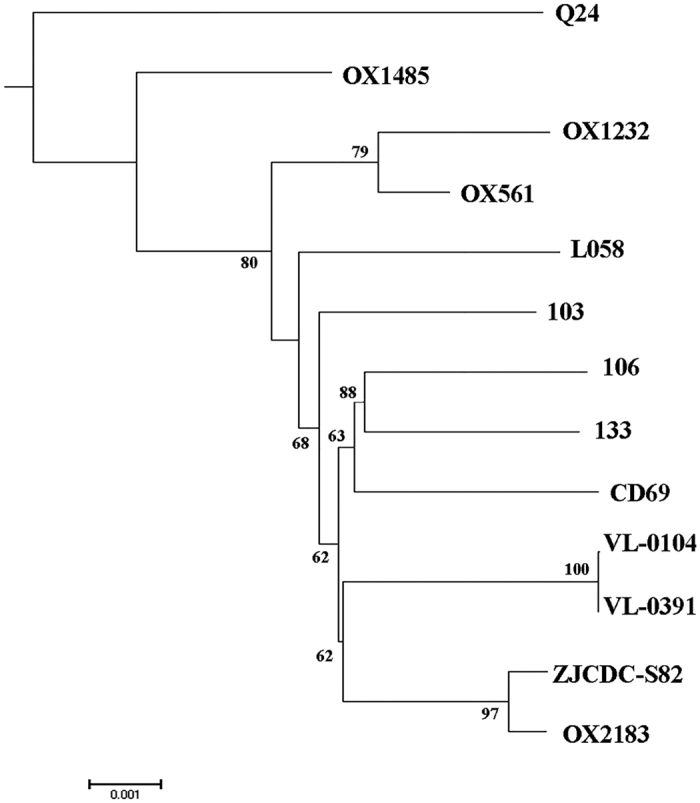
Phylogenetic tree based on SNP identified in the core genomes of Clade 3 strains. Bar, 0.01 changes per nucleotide position. Bootstrap values >50% (based on 1,000 resamplings) are shown.

All three isolates had the identical complete sequence of PaLoc and the following comparison was used the consensus PaLoc sequence of the three isolates. PaLoc of the three isolates had the same genetic organization as other Clade 3 PaLoc but with a few SNPs and insertions or deletions (Table 3). Like the PaLoc of all other Clade 3 strains, those of the three isolates had the insertion of Tn*6218* between genes *tcdE* and *tcdA* (Fig. 1). Tn*6218* in Clade 3 PaLoc had four common genes (*int, xis, rep* and *xre*) and five accessory genes, *i.e., merR* (a transcription regulator gene), a genes encoding oxidoreductase, a gene encoding flavodoxin, an open reading frame (orf) encoding a hypothetical protein containing cupin domain, and a gene encoding RNA polymerase σ70 (Fig. 1 and Table 3).

The toxin B gene *tcdB* of the three isolates were highly similar to those of all other Clade 3 strains with only 2 to 12 SNPs, but they were more distinct from those of other clades as there were 92 to 446 SNPs compared to the representative strains of Clade 1, 2 and 5 (Table 3 and Figure S3 in the Supplementary Data). Toxin A gene *tcdA* of the three isolates had 1 to 5 SNPs from several Clade 3 strains (such as OX561) but had a number of nucleotide insertions or deletions when compared with those of other Clade 3 strains such as strains CD69 and ZJCDC-S82 (Table 3 and Figure S3).

Compared with the typical PaLoc of strain CD630 (Clade 1), *tcdC* of the three isolates contained a 54-bp consecutive deletion that resulted in a truncated TcdC protein. The 54-bp deletion was also present in some Clade 3 PaLoc such as that of strain CD69 (Table 3) and Clade 1 strains AIP2005162 (GenBank accession no. EU271784), strain 55767 (GenBank accession no. FJ409552), KK296 (GenBank accession no. EF447033) and CD3980 (GenBank accession no. JN944624). In contrast, *tcdC* of other Clade 3 PaLoc such as that of strain ZJCDC-S82 had a 36-bp consecutive deletion compared with that of strain CD630.

### Sequence analysis of CdtLoc

Unlike the PaLoc of Clade 3, there were no insertions of mobile genetic elements in CdtLoc. The CdtLoc of the three isolates was identical to that of strain ZJCDC-S82 and was also highly similar to those of other Clade 3 strains. The exception to this was strain Q24 with only several SNP (Table S4 in the Supplementary Data). There were a number of SNPs between the CdtLoc of the three isolates, those of Q24 (Clade 3) and the representative strains of Clade 2 and 5 (Table S4). Phylogenetic tree of CdtLoc (Figure S4 in the Supplementary Data) revealed that the CdtLoc of all Clade 3 strains, with the exception of strain Q24, formed a cluster, while those of Q24, a Clade 5 strain and the Clade 2 strains belonged to three separate clusters.

### Prophages and plasmids

Isolate 103 contained two intact, 9 incomplete and one questionable phage, while isolate 106 did not carry any intact, but had 8 incomplete phages (Table 4). Isolate 133 contained two intact and 6 incomplete phages. Among the intact and incomplete phages, a 27.2 kb phage region was common to all of the three isolates and very similar (96.4% to 97.7% identity; 100% coverage) regions were also present on genomes of several *C. difficile* strains of other clades such as CD196 (Clade 1). The three isolates did not carry any known plasmid replicons. Among genome sequences of the three isolates, none except a 2.4 kb fragment of an 8.4 kb contig, which were seen in all of the three isolates by both SPAdes and PlasmidSPAdes, matched any sequences of known *C. difficile* plasmids. The 2.4 kb fragment matched (98% identity) with two genes of unknown function on *C. difficile* plasmid pCD6 (GenBank accession no. AY350745). The remaining part of the 8.4 kb contig did not match any known sequences on either chromosomes or plasmids of any bacteria. Therefore, whether the contig belongs to a plasmid is still questionable and needs further study.

### Unique genes

Compared with other clades, all isolates of Clade 3, including the three in the present study and those with genomic sequence available in the GenBank and SRA, had 15 unique genes (Table 5). These unique genes included 4 of the 5 accessory genes (except the gene encoding RNA polymerase σ70) of Tn*6218* in PaLoc (see above), genes encoding metallo-β-lactamase (MBL) fold metallo-hydrolyase, transcriptional regulator and methylthioadenosine/S-adenosylhomocysteine (MTA/SAH) nucleosidase, and 8 genes of unknown function.

Isolate 103 had 10 unique genes, all of which were clustered together in a 10.2 kb region of a single contig (Table 5). Isolate 106 had 12 unique genes, some of which formed two clusters (Table 5). Isolate 133 had 20 unique genes, which were clustered on either the 131.4-kb Contig 11 or the 75.0-kb Contig 24 (Table 5). None of these genes unique to each of the three isolates were predicted as part of a phage. Among the unique genes of Clade 3 and the three isolates, none belonged to an ICE and only one gene that is unique to isolate 103 (locus 103_01544; Table 5) was predicted to belong to an insertion sequence.

## Discussion

The binary toxin has been increasingly recognized as an important virulence factor. Although binary toxin genes have been found in 23% toxigenic *C. difficile* in Europe^39^, the prevalence of binary toxin genes in toxigenic *C. difficile* was less than 7% in China^14^,^19^,^20^,^21^,^22^,^23^,^24^. Such a low prevalence of binary toxin gene-carrying toxigenic *C. difficile* in China resembles the prevalence (<10%) before the emergence of hypervirulent PCR ribotype 027^17^. It has been found that binary toxin gene-carrying *C. difficile* belonged to several STs including ST1 (Clade 2), ST5 (Clade 3), ST11 (Clade 5), ST22 (Clade 3), ST201 (Clade 3) and ST220 (Clade 1) in China^7^,^19^,^20^,^40^. In the present study, we also reported a binary toxin gene-carrying strain of ST285 (Clade 3) in China. Outside of China, binary toxin gene-carrying *C. difficile* of ST5 has been reported in Oxford, UK^5^.

The estimated evolutionary rate of *C. difficile* was 0.6 to 2.3 mutations per genome per year^41^. It has been proposed that ≤2 SNPs are defined for transmission and >10 SNPs are defined for genetically distant strains^31^. There were 1,267 SNPs between isolate 103 and 133 (both of ST5), while isolate 106 (ST285) had 1,104 and 1,364 SNPs compared with isolate 133 and 103, respectively. This suggests that the three strains were not part of a common clone. Isolates 103 and 133 were recovered from the same ward, but the SNP analysis of genome sequences did not indicate the inter-patient transfer of a common strain.

ANI has been increasingly employed to determine bacterial species. The ≥95% ANI cutoff corresponds to the ≥70% DNA-DNA hybridization value^42^, which is the gold standard for defining a bacterial species. Therefore, the ≥95% ANI cutoff has been proposed to define species. Clade 3 strains had ≥95% ANI values with clades 1, 2, 4 and 5, indicating that these clades were truly of a single species. In contrast, the newly identified Clade C-I shared only 90% to 91.6% ANI values with Clade 3 strains and those of other clades (Table S2 in the Supplementary Data), suggesting that Clade C-I may represent a new bacterial species other than *C. difficile*. In addition, Clade 3 strains had >99% ANI values when compared with strains of the same clade and had <99% values with strains of other clades. This raises the question whether the >99% ANI cutoff can be employed to define clades of *C. difficile* or not. However, some strains of Clade 1 or Clade 4 had <99% ANI values with strains of the same clade (Table S2 in the Supplementary Data) and no certain ANI values were found to correctly assign all *C. difficile* strains to a clade.

The major feature of PaLoc in Clade 3 is the insertion of Tn*6218* between *tcdE* and *tcdA*, which is distinct from PaLoc in other clades. Strains belonging to Clade 3 strains were found in Australia, Bulgaria, Canada, China, UK and USA^5^,^7^,^43^, suggesting that the insertion of Tn*6218* in PaLoc is clade-specific rather than geographical location-specific. The insertion of Tn*6218* in PaLoc has also been suggested to be associated with the relatively low virulence of Clade 3^5^, as some previous studies have shown that 14-day mortality in CDI cases caused by strains of Clade 3 (PCR ribotype 023; 7%) was lower than those caused by Clade 1 (12%)^10^. However, there were additional differences between Clade 3 and Class 1 PaLoc such as SNP and/or insertions/deletions in *tcdA, tcdB* and their regulatory genes. The exact impact of Tn*6218* insertion on the virulence remains undetermined and warrants further investigations.

Previous studies suggested that Tn*6218* elements are widespread among the *C. difficile* isolates^5^. Compared to Tn*6218* elements located outside of PaLoc, those in the Clade 3 PaLoc did not carry known antimicrobial resistance genes^5^. Instead, Tn*6218* in Clade 3 PaLoc carried four accessory genes that were absent from Tn*6218* elements located outside of PaLoc, and appeared to be the main feature to distinguish Clade 3 from other *C. difficile*. In Clade 3 PaLoc, Tn*6218* is always flanked by an 863-bp sequence on one side and a 263 bp sequence on the other. The two sequences do not contain any orfs and their origins remain unclear, although 169 bp of the 863 bp sequence shows some similarity (81% identity) to several *Clostridium* phages such as phiMMP03 (GenBank accession no. LN681542). Tn*6218* requires AT rich insertion sites^5^ and the two sequences are AT rich (78% AT content for the two sequences as a whole), suggesting that the two sequences might have inserted into Clade 3 PaLoc prior to the insertion of Tn*6218* and therefore provide the insertion site of Tn*6218* like a Trojan horse. The two sequences are absent from almost all PaLoc of other clades and therefore there are no insertion sites of Tn*6218* in those PaLoc, which could explain that the insertion of Tn*6218* in PaLoc is restricted to Clade 3 so far. There is only one exception at present. In the PaLoc of strain 8864 of Clade 2 (GenBank accession no. AF035716), the two sequences are jointed together with a single copy of the 36-bp terminal repeat of Tn*6218*, suggesting that Tn*6218* might have been excised.

As for other components of PaLoc, *tcdB* appears to be more conserved in sequence than *tcdA* in Clade 3. It is well known that there are many variations in the amino acid sequence of TcdA and TcdB toxins^44^,^45^. However, the relevance of the amino acid variations of TcdA and TcdB to clinical illness remains largely undetermined as there is little evidence that the variations have significant impact on the outcome of diseases^44^,^45^. Nonetheless, a previous study has identified 8 amino acid variations in TcdB that are associated with reduced toxicity of host strains^46^. However, none of the 8 amino acid variations were present in the three isolates in the present study. Both TcdA and TcdB have four domains (glucosyltransferase, autoprotease, delivery and receptor-binding)^47^. The SNPs in *tcdA* and *tcdB* in the three isolates compared to the reference strain CD630 were distributed in all of the four domains (Figure S3 in the Supplementary Data) but their impact on the function of TcdA and TcdB remains unclear^48^, which may warrant further characterizations.

TcdC protein contains three domains. These are Hyd (Hydrophobic membrane anchor), Dim (Coiled-coil dimerization domain) and OB-fold (conserved C-terminal domain containing a predicted oligonucleotide-binding)^49^. It has been reported that mutations of *tcdC* contribute to the hypervirulence of ST1/PCR ribotype 027^50^. Compared with that of strain CD630, *tcdC* of ST1/PCR ribotype 027 has three major differences including 1) a deletion at location 117, resulting in frameshift and a truncated *tcdC* with the absence of Hyd; 2) an 18-bp deletion in the Dim-encoding region, resulting in the missing of 6 amino acids of the Dim domain; and 3) a T660A point mutation, resulting in an amino acid substitution (N219K) of the OB-fold domain (All of the above locations refer to those of *tcdC* of strain CD630). TcdC of all three isolates in the present study had two common features, which are the absence of Hyd and the N219K amino acid substitution. The two features are also seen in ST1/PCR ribotype 027. However, there was an additional deletion of 12-amino acids in the Dim domain as there is 54-bp deletion of *tcdC* in all three isolates in the present study compared with the 18-bp deletion in ST1/PCR ribotype 027. Of note, although the SNP and insertions/deletions in *tcdA, tcdB* and *tcdC* may provide useful information to untangle the evolution of PaLoc, their impact on the virulence of host strains remains undetermined. The sequence of CdtLoc appears to be more conserved than that of PaLoc. However, the sequence of CdtLoc like that of PaLoc also appears to be clade-specific. This suggests that the acquisition of CdtLoc may be prior to that *C. difficile* diverged into clades.

Among products of Clade 3-unique genes, MTA/SAH has multiple functions in bacterial metabolism and involves in quorum sensing as it produces the universal quorum sensing signal, autoinducer-2^51^. The gene encoding MBL-fold metallo-hydrolase, which is unique to Clade 3 among *C. difficile*, was also present (100 coverage, 99% identity) on the chromosome of *Clostridium symbiosum* ATCC 14940 (GenBank accession no. AWSU00000000). *C. symbiosum* is non-toxin-producing strict anaerobe as part of the human intestinal bacterial flora^52^. This suggests inter-species horizontal transfer of this gene. The exact roles of MTA/SAH and MBL-fold metallo-hydrolase in Clade 3 *C. difficile*, however, remain unclear.

The 10 unique genes of isolate 103 encoded cobalamin-binding protein, uroporphyrinogen decarboxylase (an enzyme involved in the production of heme), methylcobamide--CoM methyltransferase, iron-sulfur protein and ABC transporter proteins. It therefore appears that these genes were formed be a methionine transport system. For unique genes of isolate 106, one cluster contained 4 genes and appeared to encode a transporter, while the other cluster contained 4 genes and appeared to be associated with DNA transfer (Table 5). The unique genes on the Contig 11 of isolate 133 encoded a protein with the GIY-YIG domain and two modification methylases. The GIY-YIG domain has been implicated in a variety of cellular processes involving DNA cleavage, from self-propagation with or without introns, to the restriction of foreign DNA, to DNA repair and maintenance of genome stability^53^. The unique genes on the Contig 24 of isolate 133 encoded pilus assembly proteins and the *par* chromosome or plasmid partitioning system, raising the suspicion that this Contig may belong to an unrecognized new plasmid, although no plasmid replicon was identified. As there is no reference plasmid sequence available, long-read genome sequencing such as using PacBio may be needed to confirm whether Contig 24 truly belonged to a plasmid.

Of note, resistance to fluoroquinolones and rifampicin are usually mediated by chromosomal mutations, which are unable to be identified by ResFinder. Nonetheless, the three isolates were all susceptible to moxifloxacin and rifampicin.

## Conclusions

Although the three *C. difficile* isolates in the present study were recovered in the same hospital, it was apparent that they did not belong to a common clone. Clade 3 strains have unusual clade-specific PaLoc characteristic of Tn*6218* insertion, which appears to be the main feature to distinguish Clade 3 from other *C. difficile*. A ≥99% ANI value with a known genome of Clade 3 may be useful to assign a *C. difficile* isolate to Clade 3 without the need to construct a phylogenetic tree. Clade C-I of *C. difficile* actually appears to be a different species.

### Nucleotide sequence accession numbers

The Whole Genome Shotgun Sequencing projects of *C. difficile* isolates 103, 106 and 133 have been deposited into DDBJ/EMBL/GenBank under accession MBMH00000000, MBGC00000000 and MBGB00000000, respectively.

## Additional Information

**How to cite this article:** Chen, R. *et al*. Whole genome sequences of three Clade 3 *Clostridium difficile* strains carrying binary toxin genes in China. *Sci. Rep.* **7**, 43555; doi: 10.1038/srep43555 (2017).

**Publisher's note:** Springer Nature remains neutral with regard to jurisdictional claims in published maps and institutional affiliations.

## Supplementary Material

Supplementary File

## Figures and Tables

**Table 1:** ANI values (%) of isolates 103, 106 and 133 compared with other Clade 3 strains and representative strains of other clades.

Clade	Strain	ST	Genome accession no.	Country	Year	103 ANI value	106 ANI value	133 ANI value
3	103	5		China				
	106	285		China		99.52		
	133	5		China		99.51	99.25	
	ZJCDC-S82	5	JYNK01000000	China	2013	99.44	99.18	99.38
	OX2183	5	C00005010	UK	2009	99.43	99.16	99.36
	OX1485	5	C00004983	UK	2008	99.37	99.20	99.39
	OX561	22	C00001506	UK	2007	99.42	99.20	99.41
	OX1232	25	C00002723	UK	2007	99.46	99.18	99.49
	L058	96	C00002452	UK	2008	99.48	99.23	99.37
	Q24	162	C00006439	Australia	2008	99.43	99.23	99.28
	VL-0104	201	FAAJ01000000	Canada	NA	99.49	99.18	99.37
	VL-0391	201	FALK01000001	Canada	NA	99.48	99.28	99.36
	CD69	221	AVHE01000000	USA	2010	99.41	99.22	99.52
1	E14	2	CAMS01000000	France	NA	97.71	97.77	97.68
	CD37	3	AHJJ01000000	USA	1980	97.66	97.81	97.73
	P49	4	AVMN01000000	USA	NA	97.91	97.91	97.77
	E25	6	CAMJ01000000	France	NA	97.70	97.77	97.82
	E16	7	CAMH01000000	France	NA	97.90	97.88	97.74
	P15	8	AVLV01000000	USA	2005	97.92	97.90	97.90
	P8	10	AVLR01000000	USA	2001	97.68	97.83	97.73
	T6	13	CAMR01000000	France	NA	97.61	97.55	97.66
	P64	14	AWZL01000000	USA	2009	97.66	97.71	97.61
	5.3	15	AZSH01000000	Australia	2008	97.97	97.93	97.95
	DA00129	16	AVIY01000000	USA	2010	97.52	97.46	97.77
	CD8-15	17	LYDP01000000	Italy	2015	97.83	97.52	97.60
	P30	26	AVME01000000	USA	2009	97.63	97.50	97.57
	CD200	28	AVIF01000000	USA	2010	97.54	97.56	97.54
	P38	29	AVMH01000000	USA	2009	97.67	97.55	97.55
	P7	34	AVLQ01000000	USA	NA	97.83	97.53	97.62
	P29	35	AVMD01000000	USA	2008	97.64	97.44	97.62
	E12	42	CAMZ01000000	France	NA	97.94	97.93	97.84
	DA00126	43	AVIW01000000	USA	2010	97.72	97.78	97.77
	CD45	44	AVGY01000000	USA	2010	97.60	97.65	97.67
	CD113	45	AVHN01000000	USA	2010	97.76	97.64	97.64
	ATCC43255	46	CM000604	Canada	1935	97.77	97.78	97.73
	CD22	48	AVGP01000000	USA	2010	97.73	97.82	97.60
	T17	49	CAMT01000000	France	NA	97.98	97.74	97.76
	P11	53	AVLT01000000	USA	NA	97.89	97.82	97.80
	CD630	54	CP010905	Switzerland	1982	97.94	97.65	97.66
	T19	55	CANA01000000	France	NA	98.09	97.82	97.87
	P46	58	AVML01000018	USA	2009	97.76	97.77	97.71
	E7	63	CAMV01000000	France	NA	97.75	97.73	97.63
	Y343	83	AVLG01000000	USA	2010	97.81	97.89	97.77
	CD105KSE11	107	FJVK01000000	UK	NA	98.05	97.77	97.22
	DA00216	110	AVJX01000000	USA	2010	97.82	97.65	97.71
	DA00211	139	AVJU01000000	USA	2010	97.87	97.47	97.52
2	CD196	1	NC_013315	France	1985	97.89	97.66	97.76
	E19	62	CAMO01000000	France	2012	97.94	97.59	97.79
	T23	67	CAMN01000000	France	NA	97.79	97.71	97.84
	E15	95	CAMM01000000	France	NA	97.51	97.54	97.58
	P59	123	AVMQ01000000	USA	2009	97.57	97.58	97.59
4	M68	37	NC_017175	UK	2006	97.59	97.58	97.48
	CF5	86	FN665652	Belgium	1995	97.78	98.08	97.85
	CD002	170	CAMG01000000	France	2010	97.74	98.06	97.71
	6503	320	ADEI01000000	USA	2009	97.76	97.84	97.38
5	M120	11	NC_017174	UK	2007	96.31	96.33	96.32
C-I	CD105KSO8	297	FJUI01000000	UK	NA	91.24	91.43	90.45

NA, not available.

**Table 2:** General features of the three genomes.

Isolate	ST	Clean reads	Genome size (bp)	GC content	No. of contigs	No. of contigs ≥1,000 bp	No. of coding sequences	No. of tRNA genes
103	5	5,703,662	4,060,612	28.56%	243	69	3,777	43
106	285	5,698,996	4,080,409	28.74%	303	61	3,764	42
133	5	5,696,658	4,181,327	28.38%	276	75	4,000	43

**Table 3:** PaLoc of isolates 103, 106 and 133 compared with other Clade 3 strains and a representative strain of each Clade 1, 2 and 5.

Clade	Strain	*tcdR*	*tcdB*	*tcdE*	Tn*6218*									*tcdA*	*tcdC*
					*int*	*xis*	*rep*	*xre*	*merR*	oxidoreductase	flavodoxin	orf	*σ70*		
1	CD630	12 (6NS)	92 (48NS)	5 + 31nt del + 1nt in (7NS + 10 del)	—	—	—	—	—	—	—	—	—	116 (44NS)	5 + 318nt del (1NS + 106 del)
2	CD196	12 (6NS)	446 (175NS)	3 + 31nt del + 1nt in (6NS + 10 del)	—	—	—	—	—	—	—	—	—	89 + 63nt del (34NS + 21 del)	4 + 36nt del (12 del)
5	M120	18 (8NS)	173 (87NS)	4 + 42nt in (2NS + 14 in)	—	—	—	—	—	—	—	—	—	89SNP + 63nt del (44NS + 21 del)	3 + 15nt del (3NS + 5 del)^*a*^
3	ZJCDC-S82	ID	6 (3NS)	ID	ID	ID	5 (3NS)	ID	ID	ID	ID	ID	11 + 210nt in (5NS + 70 del)	18 (11NS)	36nt del (12 del)
	CD69	ID	12 (7NS)	ID	3 (4NS)	ID	5 (3NS)	ID	ID	ID	ID	ID	ID	153 (73NS)	ID
	VL-0391	ID	2 (2NS)	ID	7 (4NS)	ID	3 (3NS)	1 (1NS)	ID	1 (1NS)	1 (1NS)	ID	1 (0NS)	11 + 36nt del (5NS + 12 del)	36nt del (12 del)
	VL-0104	ID	2 (2NS)	ID	8 (4NS)	ID	3 (3NS)	1 (1NS)	ID	1 (1NS)	1 (1NS)	ID	1 (0NS)	11 + 36nt del (5NS + 12 del)	36nt del (12 del)
	L058	1 (1NS)	5 (2NS)	ID	5 (3NS)	ID	3 (2NS)	ID	ID	2 (2NS)	ID	ID	ID	8 (4NS)	ID
	OX561	1 (1NS)	6 (3NS)	ID	9 (3NS)	1 (1NS)	2 (2NS)	ID	ID	1 (1NS)	ID	ID	ID	7 + 63nt del (5NS + 21 del)	ID
	OX1232	1 (1NS)	6 (3NS)	ID	8 (3NS)	ID	2 (2NS)	ID	ID	1 (1NS)	ID	ID	ID	14 (10NS)	ID
	OX1485	1 (1NS)	5 (3NS)	ID	8 (3NS)	ID	2 (2NS)	ID	ID	1 (1NS)	ID	ID	ID	37 (21NS)	ID
	OX2183	ID	3 (3NS)	ID	ID	47nt in (9 in)	5 (3NS)	ID	ID	ID	ID	ID	ID	51 (23NS)	36nt del (12 del)
	Q24	ID	8 (4NS)	ID	7 (2NS)	1 (1NS)	2 + 108nt in (1NS + 36 in)	2 (1NS)	1 (1NS)	1 (1NS)	3 (0NS)	2 (0NS)	ID	62 (35NS)	36nt del (12 del)

The results shown in the Table are that the PaLoc sequence of isolate 103, 106 and 133 queries those of strains listed in the Strain column. The number of SNP is shown in the column with the resulted amino acid substitutions and deletions being indicated in parentheses. ID, identical; in, insertion; del, deletion; -, absent; nt, nucleotide; NS, non-synonymous SNP. The nucleotide deletions and insertions are consecutive. ^a^*tcdC* of strain NAP08 has a nonsense mutation, resulting in 37 aa deletions of the C-terminal end compared to those of isolates 103, 106 and 133.

**Table 4:** Predicted phages in the three isolates.

Phage region	Region length	Completeness	Total no. of orf	Mostly matched Phage^*a*^	GC %
Isolate103					
1	27.2 kb	Incomplete	30	phiCDHM19_NC_028996(11)	27.9
2	18.3 kb	Incomplete	25	phiC2_NC_009231(6)	28.3
3	24.7 kb	Questionable	27	phiCD505_NC_028764(11)	28.3
4	17.4 kb	Incomplete	22	phiCD27_NC_011398(6)	27.2
5	15.1 kb	Incomplete	10	phiCD505_NC_028764(2)	28.8
6	7.3 kb	Incomplete	12	phiCDMH11_NC_029001(4)	28.3
7	8 kb	Incomplete	9	vB_Bans_Tsamsa_NC_023007(2)	29.3
8	19.2 kb	Incomplete	9	c_st__NC_007581(6)	27.4
9	23.6 kb	Incomplete	32	phiMMP02_NC_019421(10)	27.7
10	13.8 kb	Incomplete	21	CDMH1_NC_024144(8)	27.5
11	36.1 kb	Intact	51	phiMMP03_NC_028959(23)	32.1
12	39.4 kb	Intact	61	CDMH1_NC_024144(16)	33.8
Isolate106					
1	27.2 kb	Incomplete	30	phiCDHM19_NC_028996(11)	28.04
2	26.5 kb	Incomplete	34	phiMMP03_NC_028959(11)	29.86
3	8.3 kb	Incomplete	9	vB_Bans_Tsamsa_NC_023007(2)	29.19
4	21.9 kb	Incomplete	9	c_st__NC_007581(3)	26.62
5	18.5 kb	Incomplete	22	CDMH1_NC_024144(12)	30.13
6	15.1 kb	Incomplete	11	phiCD505_NC_028764(2)	28.86
7	12.5 kb	Incomplete	21	phiMMP02_NC_019421(15)	29.12
8	25.1 kb	Incomplete	13	phiARI0746_NC_031907(3)	39.23
Isolate133					
1	27.2 kb	Incomplete	29	phiCDHM19_NC_028996(11)	28
2	32.8 kb	Incomplete	27	phiCD27_NC_011398(6)	28
3	10 kb	Incomplete	15	phiMMP02_NC_019421(7)	28.8
4	22 kb	Incomplete	10	c_st__NC_007581(6)	26.6
5	55.9 kb	Intact	59	phiCD6356_NC_015262(40)	28.4
6	21.2 kb	Incomplete	10	phiCD505_NC_028764(2)	28.8
7	18.1 kb	Incomplete	24	phiMMP02_NC_019421(14)	28.5
8	166.8 kb	Intact	231	phiCDHM19_NC_028996(46)	30

^a^The phage (phage name_GenBank accession no.) with the highest number of orf most similar to those in the region being indicated in parentheses.

**Table 5:** Unique genes of Clade 3 and isolates 103, 106 and 133.

Locus	Product^a^	Size^b^	Location	Query Coverage
Clade 3^c^				
103_01388	Transcriptional regulator	184	Contig 5^c^	100%
103_01956	Methylthioadenosine/S-adenosylhomocysteine nucleosidase	181	Contig 10^c^	97%
103_02913^d^	Hypothetical protein	133	Contig 19^c^	
103_02915^d^	Putative flavodoxin	222	Contig 19^c^	100%
103_02916^d^	Putative oxidoreductase	286	Contig 19^c^	100%
103_02917^d^	MerR Transcriptional regulator	135	Contig 19^c^	100%
103_03125	Hypothetical protein	255	Contig 22^c^	
103_03128	Hypothetical protein	200	Contig 22^c^	
103_03130	Hypothetical protein	198	Contig 22^c^	
103_03132	Hypothetical protein	233	Contig 22^c^	
103_03133	Hypothetical protein	214	Contig 22^c^	
103_03507	Hypothetical protein	372	Contig 32^c^	
103_03634	Hypothetical protein	110	Contig 34^c^	
103_03635	MBL fold metallo-hydrolase	253	Contig 34^c^	100%
103_03735	Hypothetical protein	282	Contig 63	
Isolate 103				
103_01535	Cobalamin-binding protein	220	Contig 7	95%
103_01536	Uroporphyrinogen decarboxylase	288	Contig 7	75%
103_01537	Iron-sulfur protein	574	Contig 7	
103_01538	Methylcobamide–CoM methyltransferase	344	Contig 7	97%
103_01539	Uroporphyrinogen-III decarboxylase	362	Contig 7	98%
103_01540	Membrane transport protein	303	Contig 7	
103_01541	Metal ABC transporter substrate-binding protein	273	Contig 7	99%
103_01542	Methionine ABC transporter permease	222	Contig 7	100%
103_01543	ABC transporter ATP-binding protein	335	Contig 7	97%
103_01544	IS*30* family transposase	318	Contig 7	99%
Isolate 106				
106_00822	Multidrug-efflux transporter 1 regulator	275	Contig 4	
106_00823	Cytidylate kinase	204	Contig 4	100%
106_00824	MATE family efflux transporter	442	Contig 4	100%
106_00827	RNA polymerase sigma factor FliA	138	Contig 4	100%
106_01206	Antirestriction protein ArdA	167	Contig 6	98%
106_01208	Hypothetical protein	188	Contig 6	
106_01209	Nucleotidyltransferase, polymerase	294	Contig 6	98%
106_01213	Hypothetical protein	234	Contig 6	
106_02320	Endonuclease	367	Contig 14	99%
106_03618	Ribonuclease/integrase	407	Contig 35	
106_03620	Transposase	252	Contig 35	94%
106_03734	Membrane protein	716	Contig 48	98%
Isolate 133				
133_02100	GIY-YIG catalytic domain-containing protein	401	Contig 11	
133_02107	Hypothetical protein	120	Contig 11	
133_02109	Hypothetical protein	318	Contig 11	
133_02112	Hypothetical protein	102	Contig 11	
133_02117	HaeIII-like C-5 cytosine methyltransferase	340	Contig 11	97%
133_02118	BspRI-like C-5 cytosine methyltransferase	350	Contig 11	96%
133_03129	Flp pilus assembly protein CpaB	261	Contig 24	91%
133_03131	ATPase involved in chromosome partitioning/Flp pilus assembly protein, ATPase CpaE	261	Contig 24	99%
133_03131	Type II secretion system protein E/Flp pilus assembly protein, ATPase CpaF	256	Contig 24	
133_03133	Hypothetical protein	287	Contig 24	
133_03134	Hypothetical protein	138	Contig 24	
133_03137	Hypothetical protein	176	Contig 24	
133_03174	Hypothetical protein	177	Contig 24	
133_03175	Transcriptional regulator	116	Contig 24	95%
133_03177	radC-like JAB domain protein	127	Contig 24	
133_03181	Chromosome/plasmid-partitioning protein ParA	276	Contig 24	97%
133_03182	Chromosome/plasmid-partitioning protein ParB	450	Contig 24	
133_03183	Transcriptional regulator	194	Contig 24	
133_03185	Adenosine monophosphate/protein transferase VbhT	112	Contig 24	100%

^a^Annotation was curated using Protein BLAST (https://blast.ncbi.nlm.nih.gov) against non-redundant protein sequences in GenBank. ^b^Size, the number of amino acids. ^c^Loci and locations refer to those of isolate 103. ^d^These genes are accessory genes of Tn*6218*.
