# Supplementary material for: Whole genome sequences of three Clade 3 Clostridium difficile strains carrying binary toxin genes in China
Source: Sci Rep. 2017 Mar 6;7:43555. doi: 10.1038/srep43555 (PMC5337907; doi:10.1038/srep43555)
Supplement: Supplementary File [file srep43555-s1.pdf]

Supplementary data

Whole genome sequences of three Clade 3 *Clostridium difficile* strains carrying binary toxin genes in China

Rong Chen[1,2], Yu Feng[1,2], Xiaohui Wang[1,2], Jingyu Yang[1,2], Xiaoxia Zhang[1,2], Xiaoju Lü[1,2], Zhiyong Zong[1, 2, 3§]

[1]*Center of Infectious Diseases, West China Hospital, Sichuan University, Chengdu, China.*

[2]*Division of Infectious Diseases, State Key Laboratory of Biotherapy, Chengdu, China.*

[3]*Department of Infection Control, West China Hospital, Sichuan University, Chengdu, China.*

[§]Corresponding author. Mailing address: Center of Infectious Diseases, West China Hospital (Huaxi), Guoxuexiang 37, Chengdu 610041, China. Phone: 86-28-8542-2637. Fax: 86-28-8542-3212. E-mail: zongzhiy@scu.edu.cn

Table S1. *C. difficile* plasmids with sequence available in GenBank

Plasmid name	Strain	GenBank accession no.
Unnamed	BI1	FN668943
pCDBI1	BI1	FN668942
pCD6	CD6	NC_005326
Unnamed	ATCC9689 (DSM 1296)	CP011969
pZJCDC-S82	ZJCDC-S82	JYNK01000020
p630	CD630	NC_009089

Table S2. ANI values (%) between *C. difficile* representative strains.

Clade	Strain	E14 (Clade 1)	CD196 (Clade 2)	M68 (Clade 4)	M120 (Clade 5)	CD105KSE08 (Clade C-I)
3	103	97.71	97.88	97.59	96.31	91.24
	106	97.77	97.82	97.58	96.33	91.43
	133	97.68	97.86	97.48	96.32	91.28
	ZJCDC-S82	97.64	97.75	97.44	96.24	91.38
	CD69	97.76	97.78	97.47	96.24	91.21
	VL-0391	97.77	97.82	97.53	96.28	91.38
	VL-0104	97.78	97.82	97.55	96.29	91.59
	OX561	97.81	97.78	97.41	95.98	91.26
	OX2183	97.95	97.68	97.43	96.14	91.32
	OX1485	97.74	97.80	97.45	96.00	91.27
	OX1232	97.65	97.83	97.44	95.98	91.23
	L058	97.83	97.81	97.42	96.14	91.30
	Q24	97.56	97.70	97.40	95.97	91.39
1	E14		98.68	97.60	95.91	90.94
	CD37	99.05	98.71	97.71	95.63	90.97
	P49	99.20	98.80	97.72	96.00	91.04
	E25	99.06	98.69	97.52	95.76	90.84
	E16	98.76	98.50	97.41	95.65	90.92
	P15	99.10	98.68	97.64	95.89	91.09
	P8	98.89	98.65	97.45	95.83	91.05
	T6	99.53	98.77	97.63	95.66	90.84
	P64	99.51	98.69	97.55	95.66	90.97
	5.3	99.10	98.72	97.75	96.05	90.88
	DA00129	98.81	98.49	97.40	95.81	90.80
	CD8-15	98.90	98.39	97.59	96.05	90.95
	P30	99.01	98.37	97.60	96.06	90.91
	CD200	98.85	98.46	97.69	95.94	90.87
	P38	99.07	98.47	97.64	96.16	90.95
	P7	99.12	98.64	97.77	95.99	90.88
	P29	99.02	98.39	97.65	95.94	91.05
	E12	99.40	98.80	97.76	96.22	90.86
	DA00126	98.93	98.60	97.87	96.03	90.97
	CD45	99.07	98.44	97.58	96.25	90.84
	CD113	99.04	98.46	97.69	96.11	90.94
	ATCC43255	99.06	98.53	97.73	96.15	91.00
	CD22	99.68	98.72	97.93	96.14	90.79
	T17	99.13	98.75	97.95	96.17	91.09
	P11	99.12	98.81	97.70	95.92	91.03
	CD630	99.13	98.64	97.78	95.48	91.10
	T19	99.20	98.78	97.72	96.00	91.19
	P46	98.96	98.55	97.62	95.95	91.01

	E7	99.20	98.77	97.75	95.77	90.85
	Y343	99.07	98.73	97,70	95.88	90.93
	CD105KSE11	98.91	98.60	97.67	95.55	90.57
	DA00216	99.69	98.69	97.60	95.78	90.94
	DA00211	99.12	98.54	97.53	95.91	91.15
2	CD196	98.62		97.54	95.97	91.20
	E19	98.59	99.56	97.51	95.83	91.15
	T23	98.61	99.31	97.63	95.96	90.98
	E15	98.55	99.10	97.36	95.58	90.98
	P59	98.56	99.16	97.50	95.84	90.88
4	M68	97.73	97.54		95.77	90.99
	CF5	98.05	97.91	99.64	95.98	91.00
	CD002	97.80	97.79	99.29	95.58	90.97
	6503	97.49	97.41	98.56	95.70	90.95
5	M120	95.91	95.97	95.77		91.03
C-I	CD105KSE08	90.94	91.20	90.99	91.03	

Table S3. Numbers of SNPs in core genome for each strain using isolate 103 as the reference.

Strain	No. of SNP	Strain	No. of SNP	Strain	No. of SNP	Strain	No. of SNP
CD69	1098	CD45	15973	DA00129	16251	CD34	16433
OX2183	1146	ATCC43255	16023	T19	16259	CD37	16434
ZJCDD_S82	1167	CD200	16064	T17	16273	002_P50_2011	16485
OX1232	1245	Y343	16154	CD9	16290	CD002	16516
133	1267	P38	16177	CD18	16291	CF5	16535
VL_0391	1288	T15	16186	DA00211	16301	P7	16542
VL_0104	1290	CD105KSE11	16195	T61	16312	T23	16704
L058	1298	T6	16197	P29	16343	P59	16815
106	1365	P30	16206	CD630	16354	E19	16857
OX561	1725	CD38	16219	CD22	16373	T11	16938
Q24	1987	70_100_2010	16225	CD8-15	16385	6503	16979
OX1485	2071	CD21	16236	CD42	16387	CD196	17041
CD43	15808	CD51	16242	DA00216	16408	NAP08	43640
CD113	15911	P49	16246	DA00126	16426	CD105KSO8	127182

Table S4. CdtLoc of isolates 103, 106 and 133 compared with other Clade 3 strains and a representative strain of each Clade 2 and 5[a].

Clade	Strain	*cdtR*	*cdtA*	*cdtB*
3	ZJCDC-S82	ID	ID	ID
	CD69	222nt in (37NS+74 in)	1SNP[b] (1NS)	2SNP[c] (2NS)
	VL-0391	ID	1SNP[b] (1NS)	2SNP[c] (1NS)
	VL-0104	ID	1SNP[b] (1NS)	2SNP[c] (1NS)
	L058	ID	1SNP[b] (1NS)	ID
	OX561	ID	1SNP[b] (1NS)	ID
	OX1232	ID	ID	ID
	OX1485	ID	1SNP[b] (1NS)	ID
	OX2183	ID	1SNP[b] (1NS)	ID
	Q24	5SNP (2NS)	12SNP (6NS)	25SNP (10NS)
2	CD196	8SNP (9NS)	30SNP (17NS)	36SNP (16NS)
5	M120	12SNP+36nt in (5NS+12in)	26SNP (12NS)	49SNP (25NS)

[a]The resulted amino acid substitutions and deletions are indicated in parentheses. ID, identical; del, deletion; NS, non-synonymous SNP.

[b]The 1 SNP occurred at the same position (G116A) in *cdtA*.

[c]The 2 SNPs occurred the same positions (C628T and G2,494C) in *cdtB*.

Figure S1. Schematic representations of the CdtLoc and flanking genes. The three structures represent complete CdtLoc, incomplete CdtLoc in binary toxin-negative strains and the corresponding region in non-toxigenic strains, respectively. The 5' flanking gene *cd2602* (encoding a sensor histidine kinase), the 3' flanking gene *trpS* (encoding a tRNA ligase), binary toxin genes *cdtA* and *cdtB* and the regulator gene *cdtR* are shown.

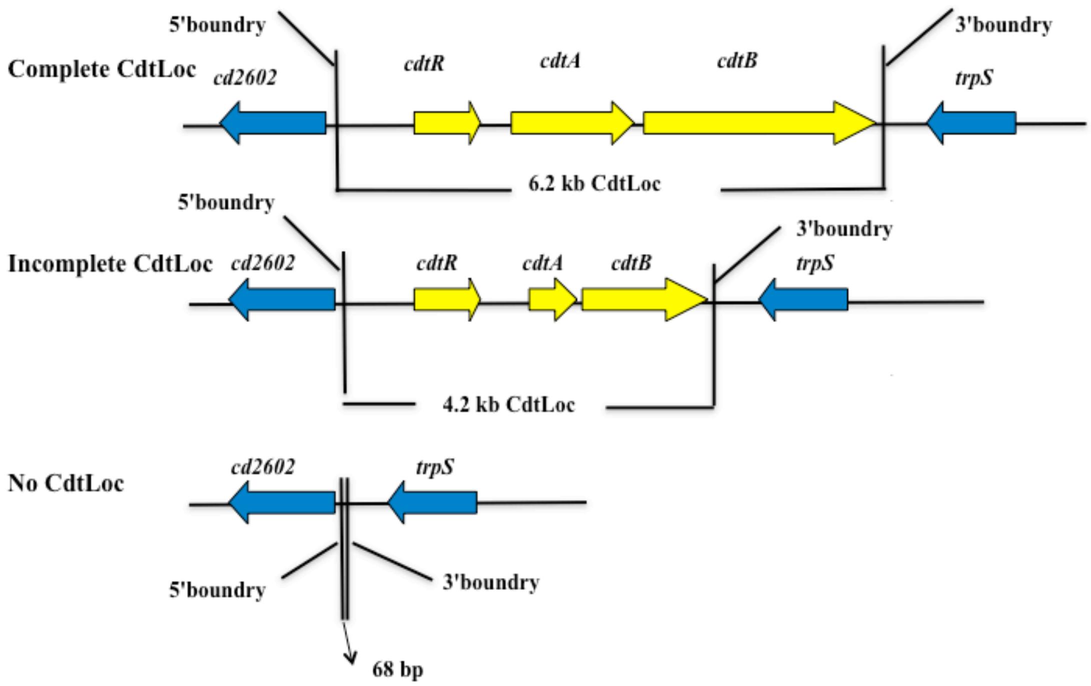

Figure S2. SNPs among the three isolates. Panel A, The triangle to show SNP between any two of the three isolates. PCR ribotype, if available, is indicated in parentheses. **Panel B,** A circular plot to show the locations of SNP of isolates 106 and 133 against the core genome of isolate 103.

A

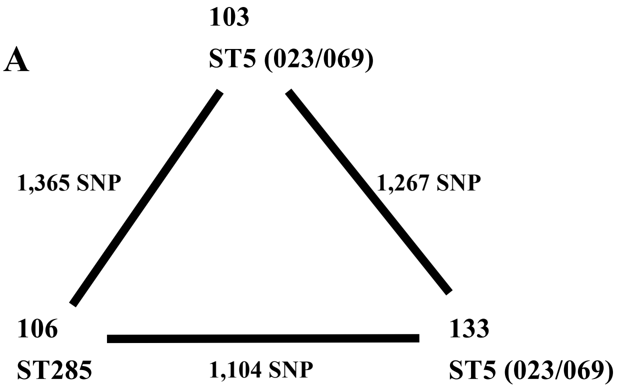

B

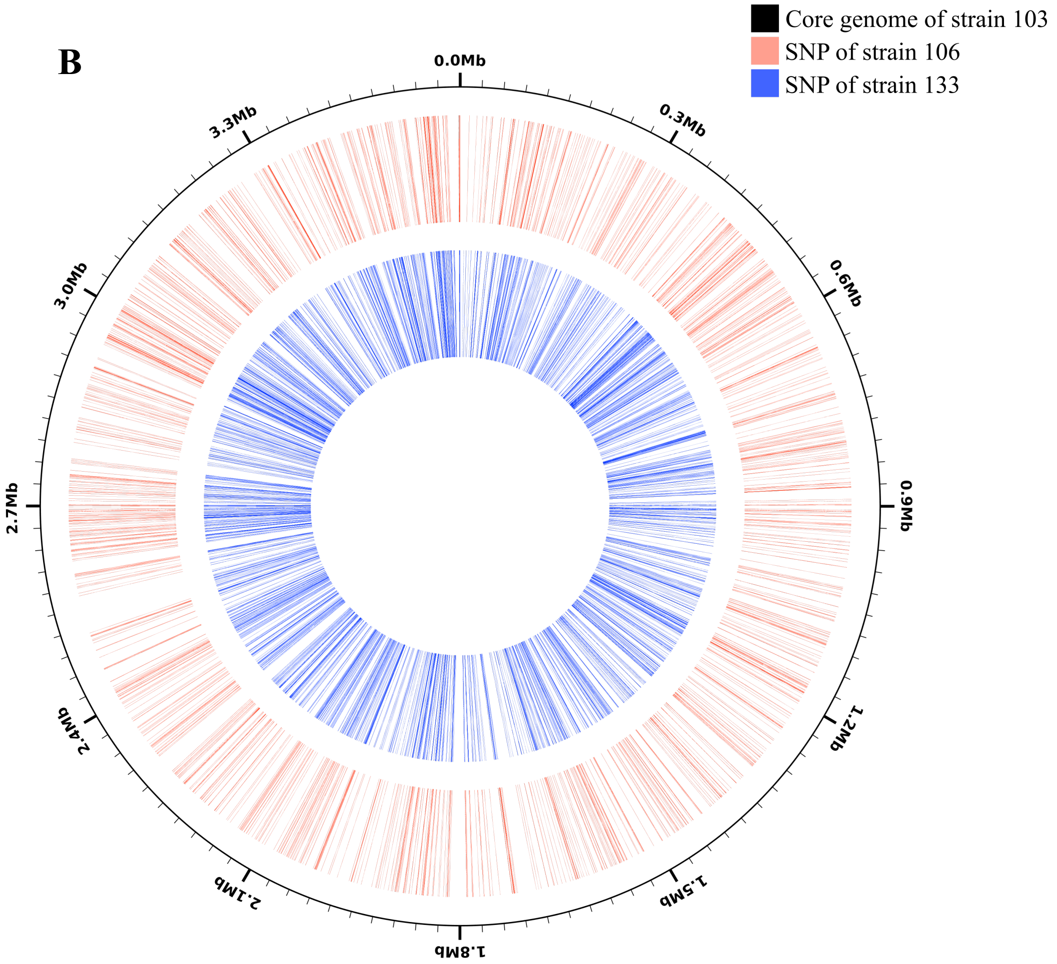

Figure S3. SNP in *tcdA* and *tcdB* sequences of the three isolates using CD630 as the reference. The coding regions of both genes have four domains, i.e glucosyltransferase, autoprotease, delivery and receptor-binding. Red lines represent nonsynonymous SNPs and blue lines represent synonymous SNPs. Panel A, *tcdA*. Panel B, *tcdB*. This figure is followed the style present in the reference[1].

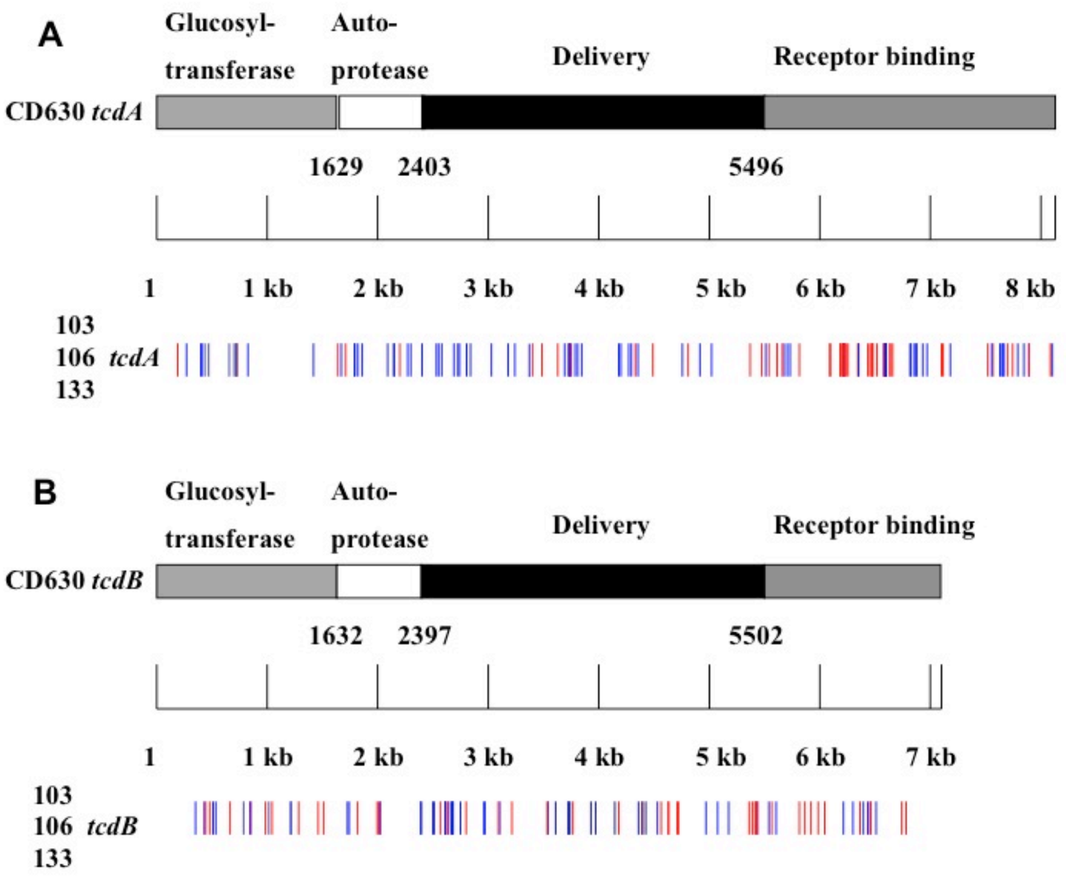

Figure S4. Phylogenetic tree based on SNPs of CdtLoc of Clade 3 strains and several representative strains of Clade 2 and 5. Bootstrap values >50% (based on 1000 resamplings) are shown. Bar, 0.001 changes per nucleotide position. The nucleotide accession numbers of CdtLoc of these strains are available in Table 1 of the Text in the manuscript.

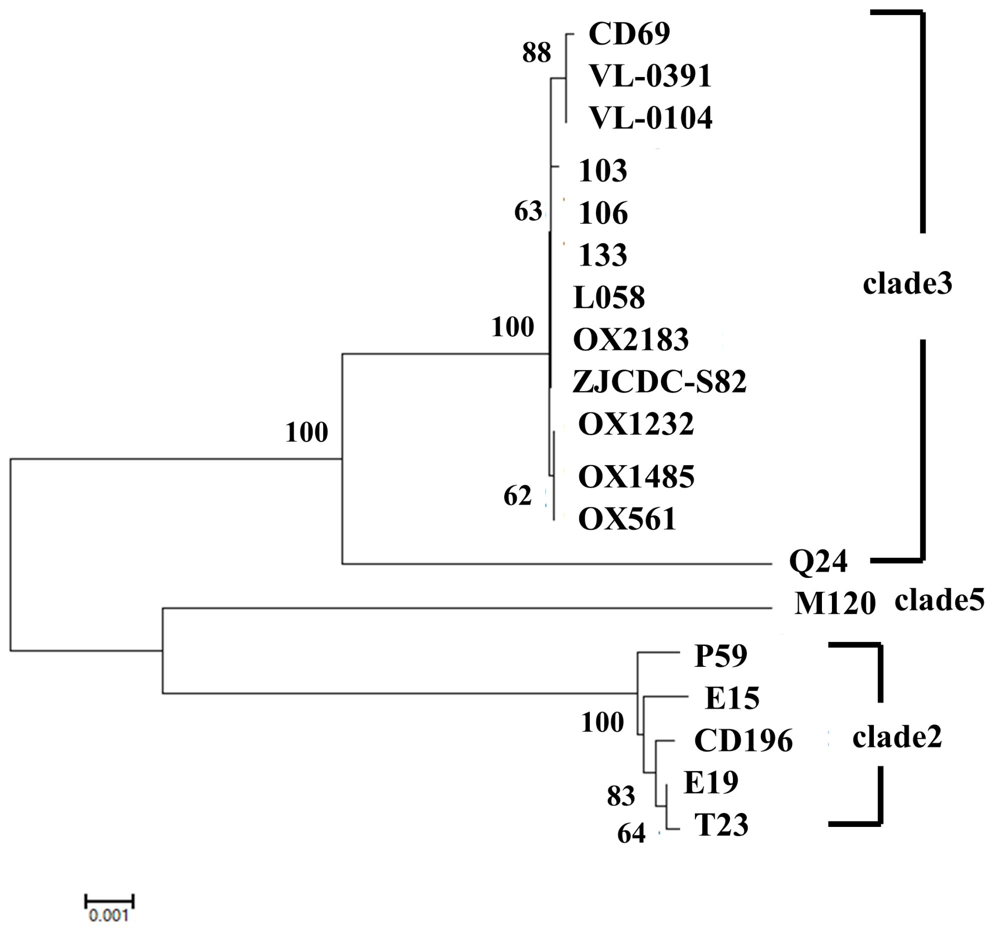

Reference

1 Du, P. *et al.* Sequence variation in *tcdA* and *tcdB* of *Clostridium difficile*: ST37 with truncated *tcdA* is a potential epidemic strain in China. *J Clin Microbiol* **52**, 3264-3270 (2014).
